# Supplementary material for: Volume and Intensity of Stepping Activity and Cardiometabolic Risk Factors in a Multi-ethnic Asian Population
Source: Int J Environ Res Public Health. 2020 Jan 30;17(3):863. doi: 10.3390/ijerph17030863 (PMC7037023; doi:10.3390/ijerph17030863)
Supplement: Supplementary file 1 [file ijerph-17-00863-s001.pdf]

Supplementary Materials

Sensitivity analyses 1: Incorporation of BMI as a covariate

Table 1. Association between step activity and cardio-metabolic risk factors (coefficients, *p* value and 95% confidence intervals)

	Step Activity Tertiles		
	**Low Reference Group**	**Moderate**	**High**
Systolic blood pressure (mmHg)		0.92 *p*=0.52 (-1.91, 3.76)	0.46 *p*=0.77 (-2.72, 3.66)
Diastolic blood pressure (mmHg)		0.92 *p*=0.37 (-1.10, 2.95)	-0.24 *p*=0.83 (-2.48, 1.99)
HDL (mmol/L)		0.01 *p*=0.70 (-0.04, 0.06)	0.04 *p*=0.09 (-0.007, 0.10)
LDL (mmol/L)		-0.11 *p*=0.17 (-0.28, 0.05)	-0.14 *p*=0.14 (-0.34, 0.05)
Triglycerides (mmol/L)		-0.03 *p*=0.66 (-0.18, 0.11)	-0.25 *p*=0.003 (-0.42, -0.09)
Fasting glucose (mmol/L)		-0.02 *p*=0.87 (-0.26, 0.22)	-0.12 *p*=0.47 (-0.45, 0.20)
Hba1c (%)		0.05 *p*=0.48 (-0.10, 0.21)	0.005 *p*=0.96 (-0.22, 0.23)

All multi variable models were adjusted for age, sex, ethnicity, education level, smoking status, alcohol use, daily average steps and BMI. Blood pressure and lipid analyses were also adjusted for medication use. Glucose and hba1c analyses were also adjusted for diagnosis of diabetes.

Table 2 Association between peak cadence and cardio-metabolic risk factors (coefficients, *p* value and 95% confidence intervals)

	**Peak 1-Minute Cadence Tertiles**			**Peak 30-Minute Cadence Tertiles**			**Peak 60-Minute Cadence Tertiles**		
	**Low** Reference group	**Moderate**	**High**	**Low** Reference group	**Moderate**	**High**	**Low** Reference group	**Moderate**	**High**
Systolic blood pressure (mmHg)		0.19 *p*=0.88 (-2.54, 2.93)	-0.53 *p*=0.76 (-4.03, 2.95)		-2.07 *p*=0.15 (-4.92, 0.78)	-0.49 *p*=0.79 (-4.36, 3.36)		-1.37 *p*=0.35 (-4.28, 1.53)	-0.39 *p*=0.84 (-4.37, 3.59)
Diastolic blood pressure (mmHg)		0.38 *p*=0.70 (-1.61, 2.39)	-1.06 *p*=0.36 (-3.35, 1.23)		-1.24 *p*=0.23 (-3.27, 0.79)	-2.74 *p*=0.04 (-5.42, -0.07)		-0.11 *p*=0.91 (-2.14, 1.92)	-1.84 *p*=0.19 (-4.63, 0.94)
HDL (mmol/L)		-0.01 *p*=0.49 (-0.07, 0.03)	0.02 *p*=0.51 (-0.04, 0.08)		-0.01 *p*=0.69 (-0.06, 0.04)	0.01 *p*=0.63 (-0.05, 0.08)		0.001 *p*=0.96 (-0.05, 0.05)	0.02 *p*=0.56 (-0.05, 0.10)
LDL (mmol/L)		0.08 *p*=0.32 (-0.08, 0.26)	0.09 *p*=0.39 (-0.11, 0.30)		0.002 *p*=0.97 (-0.17, 0.18)	0.05 *p*=0.67 (-0.19, 0.29)		0.09 *p*=0.28 (-0.08, 0.27)	0.01 *p*=0.90 (-0.23, 0.26)
Triglycerides (mmol/L)		-0.02 *p*=0.72 (-0.18, 0.13)	-0.05 *p*=0.56 (-0.22, 0.12)		0.02 *p*=0.75 (-0.13, 0.18)	-0.02 *p*=0.80 (-0.22, 0.17)		0.02 *p*=0.73 (-0.13, 0.18)	-0.05 *p*=0.60 (-0.24, 0.14)
Fasting glucose (mmol/L)		-0.37 *p*=0.01 (-0.67, -0.07)	-0.23 *p*=0.22 (-0.60, 0.14)		-0.07 *p*=0.52 (-0.32, 0.16)	-0.14 *p*=0.46 (-0.51, 0.23)		-0.14 *p*=0.26 (-0.40, 0.11)	-0.05 *p*=0.75 (-0.40, 0.29)
Hba1c (%)		-0.22 *p*=0.04 (-0.44, -0.008)	-0.16 *p*=0.21 (-0.43, 0.09)		0.01 *p*=0.89 (-0.14, 0.16)	-0.03 *p*=0.79 (-0.28, 0.22)		-0.01 *p*=0.85 (-0.18, 0.15)	0.07 *p*=0.48 (-0.13, 0.27)

All multi variable models were adjusted for age, sex, ethnicity, education level, smoking status, alcohol use, daily average steps and BMI. Blood pressure and lipid analyses were also adjusted for medication use. Glucose and hba1c analyses were also adjusted for diagnosis of diabetes.

Table 3 Association between time at 0-steps/minute and cardio-metabolic risk factors (coefficients, p value and 95% confidence intervals)

	**0-Steps/Minute Tertiles**		
	**Low**	**Moderate**	**High Reference Group**
Systolic blood pressure (mmHg)	0.13 p=0.92 (-2.70, 2.96)	0.13 p=0.92 (-2.49, 2.75)	
Diastolic blood pressure (mmHg)	1.67 p=0.09 (-0.26, 3.61)	0.44 p=0.62 (-1.35, 2.24)	
HDL (mmol/L)	-0.009 p=0.74 (-0.06, 0.04)	-0.02 p=0.34 (-0.07, 0.02)	
LDL (mmol/L)	0.13 p=0.12 (-0.03, 0.30)	0.07 p=0.34 (-0.08, 0.23)	
Triglycerides (mmol/L)	-0.003 p=0.96 (-0.14, 0.14)	-0.004 p=0.95 (-0.14, 0.13)	
Fasting glucose (mmol/L)	-0.08 p=0.62 (-0.40, 0.24)	-0.17 p=0.26 (-0.48, 0.13)	
Hba1c (%)	-0.01 p=0.90 (-0.25, 0.22)	-0.13 p=0.18 (-0.32, 0.06)	

All multi variable models were adjusted for age, sex, ethnicity, education level, smoking status, alcohol use, daily average steps and BMI. Blood pressure and lipid analyses were also adjusted for medication use. Glucose and hba1c analyses were also adjusted for diagnosis of diabetes.

Sensitivity analysis 2: Removal rather than adjustment of participants taking blood pressure or lipid medication or with a diagnosis of diabetes

Table S4. Association between step activity and cardio-metabolic risk factors (coefficients, *p* value and 95% confidence intervals).

	Step activity tertiles		
	**Low Reference Group**	**Moderate**	**High**
Systolic blood pressure (mmHg)		1.33 p=0.41 (-1.90, 4.56)	1.12 p=0.54 (-2.53, 4.79)
Diastolic blood pressure (mmHg)		0.75 p=0.52 (-1.59, 3.10)	-0.13 p=0.91 (-2.65, 2.39)
HDL (mmol/L)		0.02 p=0.45 (-0.03, 0.08)	0.05 p=0.11 (-0.01, 0.12)
LDL (mmol/L)		-0.09 p=0.29 (-0.28, 0.08)	-0.18 p=0.09 (-0.39, 0.03)
Triglycerides (mmol/L)		-0.06 p=0.44 (-0.23, 0.10)	-0.26 p=0.006 (-0.45, -0.07)
Fasting glucose (mmol/L)		-0.04 p=0.58 (-0.22, 0.12)	-0.01 p=0.92 (-0.25, 0.23)
Hba1c (%)		-0.0006 p=0.99 (-0.11, 0.11)	0.05 p=0.60 (-0.14, 0.24)

All multi variable models were adjusted for age, sex, ethnicity, education level, smoking status, alcohol use and daily average steps.

Table S5 Association between peak cadence and cardio-metabolic risk factors (coefficients, *p* value and 95% confidence intervals)

	Peak 1-minute cadence tertiles			Peak 30-minute cadence tertiles			Peak 60-minute cadence tertiles		
	**Low** Reference group	**Moderate**	**High**	**Low** Reference group	**Moderate**	**High**	**Low** Reference group	**Moderate**	**High**
Systolic blood pressure (mmHg)		-1.20 *p*=0.45 (-4.34, 1.93)	-2.83 *p*=0.15 (-6.75, 1.08)		-4.20 *p*=0.01 (-7.52, -0.88)	-3.53 *p*=0.11 (-7.87, 0.80)		-3.77 *p*=0.02 (-7.16, -0.38)	-3.73 *p*=0.10 (-8.24, 0.78)
Diastolic blood pressure (mmHg)		-0.12 *p*=0.91 (-2.40, 2.15)	-2.23 *p*=0.09 (-4.85, 0.39)		-2.56 *p*=0.03 (-4.90, -0.21)	-4.80 *p*=0.001 (-7.73, -1.87)		-1.63 *p*=0.17 (-3.96, 0.69)	-4.01 *p*=0.01 (-7.06, -0.96)
HDL (mmol/L)		0.002 *p*=0.94 (-0.05, 0.06)	0.07 *p*=0.05 (-0.0002, 0.14)		0.05 *p*=0.08 (-0.008, 0.12)	0.10 *p*=0.01 (0.02, 0.18)		0.07 *p*=0.03 (0.005, 0.13)	0.11 *p*=0.009 (0.02, 0.20)
LDL (mmol/L)		0.08 *p*=0.34 (-0.09, 0.27)	0.10 *p*=0.34 (-0.11, 0.31)		0.007 *p*=0.93 (-0.17, 0.19)	0.06 *p*=0.63 (-0.18, 0.30)		0.12 *p*=0.18 (-0.06, 0.31)	0.04 *p*=0.70 (-0.19, 0.29)
Triglycerides (mmol/L)		-0.05 *p*=0.53 (-0.22, 0.11)	-0.16 *p*=0.07 (-0.34, 0.01)		-0.14 *p*=0.08 (-0.30, 0.01)	-0.20 *p*=0.04 (-0.41, -0.002)		-0.12 *p*=0.14 (-0.28, 0.04)	-0.21 *p*=0.03 (-0.42, -0.01)
Fasting glucose (mmol/L)		-0.30 *p*=0.02 (-0.56, -0.04)	-0.24 *p*=0.12 (-0.57, 0.07)		-0.03 *p*=0.68 (-0.22, 0.14)	-0.19 *p*=0.15 (-0.47, 0.07)		-0.11 *p*=0.19 (-0.29, 0.05)	-0.08 *p*=0.48 (-0.32, 0.15)
Hba1c (%)		-0.18 *p*=0.05 (-0.38, 0.001)	-0.17 *p*=0.13 (-0.40, 0.05)		0.006 *p*=0.91 (-0.11, 0.13)	-0.10 *p*=0.29 (-0.29, 0.09)		-0.06 *p*=0.27 (-0.18, 0.05)	-0.10 *p*=0.85 (-0.16, 0.14)

All multi variable models were adjusted for age, sex, ethnicity, education level, smoking status, alcohol use and daily average steps.

Table S6. Association between time at 0-steps/minute and cardio-metabolic risk factors (coefficients, *p* value and 95% confidence intervals).

	**0-steps/minute tertiles**		
	**Low**	**Moderate**	**High Reference group**
Systolic blood pressure (mmHg)	0.38 p=0.80 (-2.66, 3.43)	0.54 p=0.71 (-2.37, 3.47)	
Diastolic blood pressure (mmHg)	1.64 p=0.13 (-0.50, 3.80)	0.20 p=0.83 (-1.81, 2.23)	
HDL (mmol/L)	-0.04 p=0.20 (-0.10, 0.02)	-0.03 p=0.21 (-0.09, 0.02)	
LDL (mmol/L)	0.10 p=0.27 (-0.08, 0.28)	0.07 p=0.37 (-0.09, 0.24)	
Triglycerides (mmol/L)	0.06 p=0.39 (-0.08, 0.22)	-0.007 p=0.92 (-0.15, 0.13)	
Fasting glucose (mmol/L)	-0.25 p=0.06 (-0.52, 0.01)	-0.28 p=0.02 (-0.54, -0.03)	
Hba1c (%)	-0.16 p=0.11 (-0.35, 0.03)	-0.17 p=0.04 (-0.35, -0.005)	

All multi variable models were adjusted for age, sex, ethnicity, education level, smoking status, alcohol use and daily average steps
